# University of Buffalo

**Published:** 1899-05

**Authors:** 


### UNIVERSITY OF BUFFALO.

### ANNUAL COMMENCEMENT OF MEDICAL, PHARMACAL AND DENTAL DEPARTMENTS.

THE 53d annual commencement of the department of medicine, the 12th annual commencement of the department of pharmacy and the 7th annual commencement of the department of dentistry of the University of Buffalo were held on Tuesday, April 25, 1899, which should properly be termed University day. Considered as a whole, the exercises this year possessed more than usual interest, especially so far as the alumni association was concerned which, as usual, led off with a morning session. In order, however, to present a compact and yet detailed report of the proceedings of

University day we shall take up for consideration the commencement exercises proper, which were observed at Music Hall in the evening.

COMMENCEMENT EXERCISES AT MUSIC HALL.

The official ceremonies pertaining to conferring the degrees were slated to begin at 7.30 o'clock p. m., but long before that time the great auditorium was thronged with the friends of the graduates, the alumni association and the university. Soon afterward the candidates, attired in cap and gown, occupied seats reserved near the stage. The members of the faculty were seated on the stage. The Hon. James O. Putnam, chancellor of the university, was unable to be present and George Gorham, acting in his stead, conferred the degrees.

After several selections by the orchestra, Prof. Herbert M. Hill presented to the acting chancellor the candidates for the degree of doctor of medicine. Prof. Mann then administered to the class the Hippocratic oath, and, amid the applause of the audience, the degrees were conferred by Mr. Gorham upon the following-named :

Lloyd L. Ackley, William W. Bachman. Samuel B. Baker, Louis J. Beyer, Frank Parker Bingham, Ph.B., Avery Kirk Brodie, Caroline W. Coats, M. D., Frances J. Coleman, M. D., Robert Edward DeCue, Bernard F. Dennis, Leo. Joseph Doll, Martin Joseph Downey, W. Levell Draper, M. D., J. Fred Eckerson, Frank X. Fiegel, James P. G. Gould, Guy L. Granger, William Walter Grantier, Edward Parker Hay, Thomas Marsden Heard, Jr., Myrtle A. Hoag, Mary L. Jennings, Charles Kelley, Ph. G., Catharine E. Kelley, Frank H. Lattin, Gerra K. Lester, Ira Warner Livermore, Arthur McCarthy, George F. Mills, Francis M. O'Gorman, William Nelson Pette, Francis N. Pitass, Guy E. Ridgway, Julien Arthur Riester, Charles C. Roosa, Patrick J. Ryan, Isadore J. Saylin, Edward A. Schweigert, Henry J. Siedler, Ph. G., John Arthur Spengler, B. L., M. L., B. S., George S. Staniland, Rae Latham Strong, Charles St. John, Ph. G., Dean O. Thompson, William Irving Thornton, Alfred F. Zittel.

Dr. Gregory announced the honor roll as follows: Frank X. Fiegel, Arthur McCarthy, Myrtle A. Hoag, Frank Parker Bingham, Ph. B., Ira Warner Livermore and Robert Edward DeCue.

Miss Catharine E. Kelley's diploma is withheld temporarily owing to the fact that she is not yet 21 years old.

The candidates for the degree of graduate in pharmacy were presented by Dr. John R. Gray.

The degree of master of pharmacy was conferred upon the following-named in accordance with the recommendation of the faculty: S. Hobart Dorr, Ph. G., John P. Meidenbauer, Ph. G., Melvin McAlone, Louise F. Morris, Ira H. Watson, Nelson McKay Wiegand, William H. Wood.

The degree of graduate in pharmacy was conferred upon Clifford B. Anthony, Herbert M. Anthony, Joseph P. Corbett, Harry H. Coulson, James J. Dargan, Frank T. Dewey, Herbert R. Edmonds, Warren F. Gardner, Ella M. Garlick, Harry M. Gates, Edward W. Hodson, Burr R. Hollands, Lucius E. Ingersoll, Mary R. Jenkins, Maurice M. Kinsey, Ellis T. Lathbury, William E. Lemon, George W. Lindner, Harry C. Luke, Henry T. Monroe, Rose M. Norton, John C. Peterson, John H. Rider, Horace G. Stilwell, Amos H. Thayer, Abram L. Weil, M. D.

William H. Wood, Melvin McAlone, Frank T. Dewey, Harry M. Gates and Lucius E. Ingersoll were on the honor roll. The Peabody prize was carried off by William H. Wood and Miss Louise F. Morris won the Alumni Association prize.

Dr. Snow presented the candidates for the degree of doctor of dental surgery. They were: Frank Anderson, George Morris Austin, William George Beaumont, Charles Alonzo Bennett, Frank Joseph Biker, Michael Courtney Bradley, William John Burke, Duncan Alexander Cant, Frederic William Champlin, Charles Hoyt Churchill, Arthur Burdette Cobb, Frank Winthrop Cook, Frederic Seaman Cox, Leon Van Cursons, Guy Rufus Danforth, George Edward Dougall, John Elmer Dunn, Harry Gardiner Fairfield, Robert Joseph Fletcher, Charles Joseph Fraley, Charles Elisha Gillam, Gladstone Goode, Bertrand Oscar Harmon, Charles Henry Hickleton, Abram Hoffman, George Lee Horton, Arthur Fuller Isham, William Dawson Jacob, Alfred Osman Jerrett, Arthur John Jessel, Clement Dale Kennedy, Burt Kinsella, Francais Montgomery Lee, William Nicklis Leonard, Harry Hethcote Luton, Harry Koch Mason, Russel George William Merkley, Stanley Albert Merkley, John Middaugh, George Franklin Mouthrop, Mansfield Earnest Mooney, Arthur Bryce Muir, Emanuel Muntz, Daniel McPherson Murray, Thomas Francis O'Shea, George O'Leary, Frederick William Orwan, Herman Eugene Reynolds, Clare Eugene Robinson, William James Roche, Robert Sabin, Robert Roy Schmidt, Henry Hugo Adolph Semtner, Grace Newton Shirley, B. L., Howard Arthur Smith, Louis Weston Smith, Alonzo Whitcomb Tracy, James Forbes Wardner, William Howard Willson.

The diploma of Leon Van Cursons was withheld because of the fact that he was under age. It will be delivered as soon as he attains his majority.

The names on the honor roll were Arthur John Jessel, Arthur Fuller Isham, Abram Hoffman, Grace Newton Shirley, B. L. Gladstone Goode and Charles Elisha Gillam.

The annual address to the graduates was delivered by the Rev. Henry Elliott Mott, D. D., who also pronounced the benediction.

Three banquets followed the commencement exercises. The medical department banquet took palce in the banquet room at Music Hall, an account of which appears in its appropriate place.

The pharmacy and dentistry departments held their banquets at the Ellicott Club. At the dentists' dinner, held in the large dining room, the toast list was as follows: Divine Elucidators of Toothotomy, Prof. Geo. B. Snow; Our Colleges, Prof. Faneuil D. Weisse; Our Respected Jugglers of Pills and Plasters, Dr. Almon H. Cooke; Disciples of Munchausen, Charles F. Kingsley; Rag-time Talk of the Past, Dr. William J. Roche; *x*-ray Light on the Future, Dr. Emanuel Muntz; Muses that Amuse, Dr. Frank W. Cook; The Bunch, Dr. Frank W. Sibley.

Dr. Meidenbauer was toast-master at the pharmacy banquet, which was held in the ladies' cafe, and toasts were responded to as follows: The University, Dr. Lucien Howe; Pharmacy and Law, Devoe P. Hodson; Circulator Medicus et Circulator Theologicus Circumforaneus, the Rev. Henry Elliott Mott; Up San Juan Hill, Major Auman, U. S. Army; Pharmacal Education, Prof. George C. Diekman; Pharmacy and War, Dr. A. H. Briggs; Class of 1899, Ira H. Watson; Pharmacal Education, R. K. Smither.

### ALUMNI ASSOCIATION.—MORNING SESSION.

A marked feature of the morning session this year was an informal reception given by the committee of women physicians to women graduates and the wives of visiting alumni, which was held in the library of the University from 10 until 12.30 o'clock. Afterward luncheon was served and at which several felicitous little speeches were made. The library was appropriately decorated with rugs, palms, flowering plants, flags and the college colors.

The reception committee consisted of Ida C. Bender, chairman; Maud J. Frye, Mary I. Denton, Edith M. Stewart, Margaret S. Halleck, Lillian C. Randall, Jeannette P. Himmelsbach, Amelia E. Trout, Marie S. Ross, Alice Ross Bennett, Bina P. Vandenberg,

Mary M. Huntley, Cora B. Lattin, Jane W. Carroll, Carro J. Cummings, Alice L. Mitchell and Jane N. Fear.

Among the guests were: Mrs. Frank A. Jones, Rochester; Mrs. Wm. J. Howe, Scottsville; Mrs. Clara Boysen, Egg Harbor City, N. J.; Mary B. Wetmore, Buffalo; Mrs. C. M. Briggs, Fairport; Alice M. Potter, Ithaca; Mrs. Mary A. Potter, Ithaca; Mrs. A. L. Benedict, Buffalo; Harriott Sheldon, Buffalo; Mrs. Thos. Bagley, Buffalo; Charlotte Masten, Wellsburg; Helen Kennedy, Buffalo; Louise Westlake, LeRoy; Mary Hayes, Kane, Pa.; Mrs. John R. Gray, Buffalo; Hortense V. Bruce, Buffalo; Mary Clayton, Buffalo; N. Victoria Chappell, Buffalo; Miss Emma Chappell, Buffalo; Electa B. Whipple, Buffalo; Mrs. Z. J. Lusk and Miss Lusk, Warsaw; Caroline W. Coats, Francis J. Coleman, Myrtle A. Hoag, Mary L. Jennings, Katherine E. Kelly, all of the graduating class and members of the faculty.

Of those of fifty years ago who were to meet in reunion only such war horses as C. C. Wyckoff, '48, Walter D. O. K. Strong and Frederick F. Hoyer, '49, were vigorous enough to meet again as college boys after a lapse of a half century. Of 1869 four were present, and an extremely pleasant time they had reminisencing. Those present were: A. D. Atwood, Brooklyn; John H. Taylor, Holly; Frank A. Jones, Rochester, and D. G. Hubbard, Buffalo. Dr. Robert A. Patchin, Des Moines, Ia., was elected president.

Of those of 1859 only two responded to the roll call—Hamilton E. Smith, of Detroit, Mich., and William Warren Potter, of Buffalo. Dr. Smith was elected president and class orator for the ensuing year.

A very enthusiastic class reunion was that of 1874. Mutual recognitions were difficult and caused much amusement, for time had wrought its usual changes, but once more together they were the "old boys" and lived over again the old times. There were present fifteen members of the surviving twenty-nine, and the only saddening feature of the occasion was the thought of the seven who had gone to the great majority. Those present were: Edward N. Brush, Towson, Md.; Bernard Bartow, Buffalo; Theo. H. Boysen, Egg Harbor City, N. J.; Robt. N. Bush, Horseheads; Marcenus H. Cole, Newfane; David B. Horton, Red Creek; John A. Love, Warren, Pa.; John A. Pettit, Buffalo; Darius G. Pickett, Fredonia; Clinton A. Sage, Buffalo; John R. Selover Applegate; Adin P. Waid, Buffalo; William D. Whitney, Muncie, Ind.; William J. Howe, Scottsville, and T. L. Barry, East Aurora.

Letters of regret were received from several members. The morning was largely devoted to an experience meeting which was hugely enjoyed by all. A formal organisation was then made by selecting E. N. Brush, president and Wm. J. Howe, secretary. Upon motion of Bernard Bartow, the president appointed a committee of three, consisting of Drs. Bartow, Pettit and Horton to confer with the members of the class concerning contributing to an endowment fund for the University. The president was added to the committee by motion of Dr. Horton. It was decided that a brief history of the members of the class be compiled for publication and that a copy be sent to each member. The class then adjourned to the Genesee House for luncheon, which added very much to the pleasure of the occasion.

The class of '79 held its first reunion and the following officers were elected for the Pan-American year, 1901: President, Wm. L. Dickinson, E. Saginaw; vice-president, E. J. Drury, Phoenix; secretary, Benj. F. Rogers, Buffalo; treasurer, L. E. Ellis, Detroit; F. H. Bartlett, Bradford, Pa.

The class of '89, made a record with the following members present, including the alumni of Niagara University, '89: Lyman C. Broughton, Castile; John C. Brown, Smethport, Pa.; Wm. O. Burbank, Pavilion; Thomas Bagley, Buffalo; Elmer E. Briggs, Buffalo; Robert S. Carr, Williamson; E. A. Forsyth, Univ. Niagara, Buffalo; John R. Gray, Buffalo; Walter E. Gregory, Dansville; Harold A. Hayes, Buffalo; John T. James, Medina; J. M. Kraus, Buffalo; Frederick W. Koehler, Buffalo; Henry J. Mulford, Buffalo; Wm. Stanton, Varysburg; Wm. T. Twitty, Buffalo; Alfred W. Bayliss, Univ. Niagara, Buffalo; Washington I. Hewitt, Univ. Niagara, Olean.

Class officers for the ensuing year were elected as follows: President, Grover W. Wende, Buffalo; vice-president, Washington I. Hewitt, Univ. Niagara, Olean; secretary, Frederick Koehler, Buffalo; orator, A. W. Henckell, Rochester.

It was unanimously resolved to subscribe individually towards an endowment fund for the benefit of the medical department of the university. After an enjoyable and interesting reunion the class adjourned to meet at the alumni meeting of the university in 1900.

### AFTERNOON SESSION.

The regular session of the association was called to order in alumni hall at 2.30 p. m., by Dr. Z. J. Lusk, president. Minutes of he meetings of April 26, 1898, and February 22, 1899, were read and approved, after which the president delivered his address, which will be published later in the JOURNAL. The report of the treasurer, Dr. Herbert U. Williams, was then submitted as follows:

I beg to hand you herewith my detailed report as treasurer, showing total receipts to date, $402.11, with expenses of conducting the exercises of April 26, 1898, and our share of the alumni reunion of February 22, 1899, $394.33, leaving cash balance on hand of $7.78.

A year ago the executive committee were empowered to take such steps as they sought fit for the collecting of unpaid dues from the members, but as no scheme has been as yet devised to do the same, I would report that upon my books are registered 489 members live and active, from whom upon April 25, 1899, became due $4,571. This association has always felt the need of money and has had continually to ask for donations from those most willing to give. Could the money due the association, or a large share of it, be obtained, the financial status would be one which would warrant expansion into fields which would be of interest to each member. The number eligible to membership in this association today is about 1800. I find that each graduating class adds but few to our number. I would, therefore, recommend that the executive committee with the treasurer be empowered to collect the amounts due to the association from delinquent members, by compromise or otherwise, as in their judgment seems best fitted to each individual case, and I would recommend that in order that the graduates may be encouraged to become active members in this association that the following amendment be made to the by-laws, to-wit:

That there be added to article I. of the by-laws, adopted April 27, 1897, a section to be known as section 3, which shall read, that graduates of the medical department of the University of Buffalo who upon the date of graduation shall apply for membership in this association shall have his annual dues in said association remitted for the year next following the year of his graduation. By this amendment dues as an active member will begin after he has been two years in active practice. I would further recommend that as active membership is the only relation which individuals should bear to this association, and as life-membership begets a lack of interest in the association, that section 11 of article I. of the by-laws, adopted April 27, 1897, and which reads, "That the member who shall pay the sum of $10 at any one time shall thereby be made a life-member and be exempt from further payment of annual dues," stand annulled from this day and date.

I sincerely hope that these recommendations may be adopted to the advantage of our association.

Respectfully submitted,

HERBERT U. WILLIAMS, Treasurer.

Dr. Lytle moved, and it was carried, that the proposed amendment be received and placed on file until the next regular meeting.

Dr. Chappell moved that the chair appoint an auditing committee. Carried.

The president appointed Drs. Bingham and Bartow, who subsequently reported that the treasurer's detailed report was correct. Their report was accepted and filed.

The report of the executive committee was then presented as follows:

For the annual meeting of April 27, 1898, we expended a sum of money equivalent to $344.33; for the reunion, held February 22, 1899, we spent the sum of $50, as shown in the treasurer's report. The committee deemed it wise to have a reunion to welcome the adopted alumni of the University of Niagara into its fold. Being without funds to warrant such an expense, we addressed the permanent faculty of the medical department and they unanimously seconded our efforts by appointing two of their number to coöperate with the executive committee, and at the same time furnished the necessary funds for the carrying out of our proposition. From the reprint mailed you, you will see that this reunion was an unqualified success. We have been encouraged to initiate ten-year class reunions, beginning with the classes as named in the annual announcement. This has required a large amount of labor, a great part of which has been performed by Miss Emma L. Chappell, who has given her services freely and without compensation, and the executive committee wish now to publicly acknowledge their indebtedness for the same.

We feel that in the enlarged field in which the association can work, certain changes should be made to the constitution and by-laws, and we therefore recommend the following amendments to the constitution, adopted April 27, 1897, to-wit:

That article IV., section 1, which now reads, "The officers of this association shall be a president, five vice-presidents, two of whom shall be residents of Buffalo, a secretary, a treasurer, a board of trustees, consisting of five members, and an executive committee, consisting of five members, two of whom shall be respectively the president of this association and the secretary of the faculty, each ex-officio," shall read, "The officers of this association shall be a president, five vice-presidents, two of whom shall be residents of Buffalo, a secretary, a treasurer, a board of trustees, consisting of five members, an executive committee, consisting of seven members, four of whom shall be respectively the president, the secretary and the treasurer of this association and the secretary of the permanent faculty of the medical department, each ex-officio, and that a section to be known as section 5, article IV., shall be added which shall read "The officers of this association shall only be chosen from the regular active members in good standing." That section 1, article VI., shall be so changed as to read "all nominations and elections shall be by ballot as hereafter provided. The members nominated and subsequently elected to office by a plurality vote of the members of the association in good standing shall enter upon their duties immediately upon the final adjournment of the annual session of the association, and said officers shall hold office for one year or until their successors are elected as hereinafter provided; and that a section to be known as section 3, article VI., be created to read "A member in good standing in this association is one whose dues are paid in full" and that there be inserted in article V., section 3, after the word "ten," section 2, and after the word "members," section 3, the words "regular active members in good standing," and after the word "members" in section 1, article VII., be inserted "in good standing."

We also recommend the following amendments to the by-laws by adding to article V. three new sections to be known as sections 5, 6, 7, which shall read as follows, to-wit:

Section 5. The executive committee shall annually and not later than March 1st, prepare and submit to each regular active member in good standing a list containing six names for each office becoming vacant, at the annual meeting next following. This list shall be known as the "nominating ballot."

The executive committee shall receive, canvass and record the casts of the nominating ballot.

Section 6. The executive committee shall annually and not later than April 1st, prepare and submit to each regular active member in good standing, a ballot to be known as the "official ballot"; said official ballot shall contain two names for each office becoming vacant at the annual meeting next following. The said names shall be those receiving the greatest number of votes for the respective offices, as shown by the cast of the nominating ballot, as provided for in section 5 of this article.

Section 7. The executive committee shall receive, canvass and record all ballots cast for the officers of this association up to 3 o'clock in the afternoon of the day of the annual meeting, at which time the polls shall be closed.

The executive committee shall report to this association not later than 5 o'clock in the afternoon of the day of the annual meeting the result of the cast of the ballots for the officers of this association.

The executive committee would further recommend that the Buffalo Medical Journal be designated as the official journal of this association and that the secretary be instructed to furnish the editors thereof with a complete report of the various meetings of this association, including the papers presented, as soon after the meetings as possible.

We further recommend that the secretary be instructed to secure sufficient reprints from said journal so that each alumnus of the medical department may receive at the next convenient time a copy thereof.

The executive committee wishes to acknowledge its indebtedness to the various members of both the men's and women's committees, under the able management of Drs. Sherman and Bender, for their efficient services.

THE EXECUTIVE COMMITEE.

By ALBERT T. LYTLE, *Chairman.*

Dr. Chappell moved and it was carried that the report be received and placed on file and that the recommendations of changes to constitution and by-laws pursue the usual course.

Dr. William C. Taylor, of the University of Niagara, then read a paper upon Prophylaxis in obstetrical practice, which with the discussion by Drs. Matthew D. Mann and others will appear in a later number of the JOURNAL.

Dr. Lytle then moved that the president appoint a committee of five to make nominations for the officers for the ensuing year. Carried. The president appointed Drs. B. G. Long, N. W. Wilson, Matzinger, Parker and Bingham.

Dr. Barrows then presented the following resolutions which were adopted upon his motion.

WHEREAS, The objects of the alumni association of the medical department of the University of Buffalo are the fostering and fraternal spirit among the graduates of the medical department of the University of Buffalo, the sustaining of interest in the medical department of the University of Buffalo, and advancement of medical education and medical science, as stated in the constitution, article II.; and

WHEREAS, In large universities like those of Harvard, Yale, Cornell and Pennsylvania, each alumnus has a voice in the management of the affairs of the university; and

WHEREAS, This participation in the management of the affairs of an institution propagates, stimulates and maintains sympathy, loyalty and interest in the welfare of an institution. Be it

*Resolved*, That this association believes that the alumni of this university should have a representative in the council of this university, chosen for a term of years by a plurality vote of the alumni in good standing. And be it

*Resolved*, That the executive committee be instructed to lay these resolutions before the permanent faculty of the medical department and before the council of the university, to demonstrate to them the reciprocal advantages which will ensue and that they report thereon at the next annual meeting.

The nominating committee then reported the following choice of officers for the ensuing year, 1899-1900: President, William Warren Potter, '59, Buffalo; 1st vice-president, Devillo W. Harrington, '71, Buffalo; 2d vice-president, Charles R. Barber, '82, Rochester; 3d vice-president, W. R. Campbell, '80, Niagara Falls; 4th vice-president, A. W. Henchell, '89, Rochester; 5th vice-president, N. Victoria Chappell, '92, Buffalo; secretary, T. H. McKee,

'98, Buffalo; treasurer, Herbert U. Williams, '89, Buffalo; trustee for five years, P. W. VanPeyma, '75, Buffalo; executive committee, A. T. Lytle, '93, Buffalo; G. W. Wende, '89, Buffalo; A. T. Kerr, Jr., '97, Buffalo.

On motion of Dr. Rochester it was ordered that the secretary cast one vote for these officers, which was done and they were declared elected.

A paper was then read by Dr. William G. Bergtold, of Denver, Col., entitled Human hemoglobin in high altitudes, which was discussed by Drs. Charles G. Stockton, Albert E. Woehnert and A. L. Benedict, which will be published later.

Dr. John H. Pryor read a paper entitled, The relative death-rates of cancer and of consumption, which was discussed by Drs. H. R. Hopkins, DeLancey Rochester, H. R. Gaylord and George E. Blackham. (To be published.)

Dr. McKee moved and it was carried, that a vote of thanks be extended to Miss Emma L. Chappell for her services to the executive committee.

Dr. Lytle then moved that the prepared amendment to article III. of the constitution be taken from the table and read. Carried. He then moved the adoption of the amendment, which reads : Every graduate of the medical department of the University of Niagara in good standing shall become a regular, active member of the association upon payment of membership dues. Carried.

Dr. Lytle moved and it was carried, that a vote of thanks be extended to Drs. Sherman and Bender and their associate committee for their excellent work upon the reception committee. He further moved and it was carried that the BUFFALO MEDICAL JOURNAL be designated the official journal of this association.

Dr. George H. Fox, of the College of Physicians and Surgeons, New York, then presented about 100 stereopticon slides, showing the many phases of cutaneous syphilis. At the same time Dr. Fox indicated in a concise manner their diagnostic features; these slides are from his large and original collection and to those listening and looking it was equal if not superior to a clinic. The reception of this lecture was enthusiastic and it was discussed by Drs. Charles Cary and Ernest Wende.

Dr. Cary moved a rising vote of thanks to Doctor Fox for the beautiful illustrations and the instructive explanations, which was carried.

Letters and telegrams of regret and good wishes were presented. A motion to adjourn was made and carried at 6 p. m.

EVENING SESSION—9.30 P. M.

The annual dinner was held in the banquet room of Music Hall, which was elaborately decorated with national colors. Alumni from 1847 to 1899 inclusive sat down to a delightful spread; music and college songs filled in the time until the toasts were announced by Edward N. Brush, as toastmaster, in his usual entertaining manner. The list was as follows: Forty-niners, '47-8-9, Walter D. O. K. Strong; Twice Two Score, '59, William Warren Potter; Endowment, Eugene A. Smith; Three Decades, '69, Frank A. Jones; A Quarter Century, '74, Robert P. Bush; A Score, '79, William L. Dickinson; Specialism, Gecrge H. Fox; A Decade, '89, Robert S. Carr; Today, '99, Frank P. Bingham; Tomorrow, Mary Innes Denton; Farewell, A Poem, Thomas M. Heard.

Dr. Potter, said with reference to the question of endowment :

You know the changes which medical teaching has undergone during the last few years and that it requires money to carry on such an institution as the University of Buffalo. You are also well aware that it is without endowment, yet there are certain teaching departments whose instructors are compelled to give their entire time to college work and are unable to devote any of it to the practice of their profession. It seems to me that it should be a pleasure as well as a duty to raise sufficient money to endow, or at least help to endow, some of these chairs. I trust that the members of the association here assembled will take the matter unto themselves seriously and each contribute within his means. It seems to me that the time has arrived when every loyal alumnus should put his hand in his pocket and give according to his ability as a pleasant duty.

Dr. Eugene Smith in substance said : Those of you who were present this afternoon will remember hearing what seemed to be a very innocent resolution to the effect that on recommendation of the alumni association the permanent faculty and the council of the university are requested to name a member from the alumni association to represent it in the council of the university. I have no doubt that it is the entering wedge of a movement which is underway to develop the so-called alumni association endowment fund. During the past few years the medical department has been expending practically every cent of its income. More, the members of the faculty, who, in bygone days were able to put some profits into their pockets are now taking those profits out, as well as adding thereto earnings made in other legitimate ways to build up the department. Each class according to its talents, the newest having the greatest enthusiasm, but least money, and vice versa, should contribute to building up the endowment fund. As to the final disposition of that fund Dr. Potter has already suggested certain channels. It can also be used for the improvement of the medical department and the benefit of the alumni by endowing special lectureships, by putting in new apparatus, necessary, nay absolutely essential, in these days of modern medicine to make it intelligible to the student, and to turn out men who will be a credit to the institution and its alumni and a benefit to suffering humanity. The other day, $2,500,000 was presented to Chicago university, which the president set aside for the benefit of one department only and he seemingly without hesitation called for $3,500,000 more for other departments. Now I am amazed to think that our university, over fifty years old has no endowment fund whatever and wonder why we have so good a school. Need I instruct medical men how they can best advance this fund without absorbing their own returns often so poorly representing their hard work? We can approach those who are well provided with this world's goods at a time when they wish to make their peace with mankind, and tell them that a clause in their will endowing the university to any amount from $5,000 to $5,000,000 would benefit their fellowmen and be an enduring monument.

In conclusion he said, that for the credit of our alma mater, for the credit of educational growth in this end of the Empire state, for the literary reputation which we wish Buffalo and this end of the state to possess, we ought earnestly to strive to make this endowment a large one, leading one class to emulate another until the $500 of the class of '89 will be $5,000 for '79, $50,000 for '59 and so on to a fund commensurate with the institution.

Dr. Frank A. Jones spoke in an interesting and humorous strain of the class of '69.

Dr. Robert P. Bush gave an interesting account of the difficulties met with in securing legislation to elevate the profession and take it from the hands of charlatans, mountebanks and quacks, he, as an assemblyman and as speaker, having been largely instrumental in securing the passage of the present laws in this state, requiring a standard education for all would-be medical students, and a standard education for all would be-practitioners. His remarks aroused enthusiastic approbation.

Dr. W. L. Dickinson, in a witty speech, told a few stories at the expense of his classmates.

Dr. George H. Fox enlivened the occasion with many anecdotes illustrative of the subject.

Dr. Robert S. Carr spoke of the efforts and success of his class in starting the alumni endowment fund.

Dr. F. P. Bingham introduced the class of '99 in an extremely funny way and received applause therefor.

One of the events upon the toast list was the response of Dr. Mary I. Denton, who clearly showed the necessity for a united effort among the alumni to throttle quackery, while she pleaded eloquently for an endowment fund for the alma mater, so sorely in need of aid to maintain her enviable position among mankind.

Dr. Thomas M. Heard read the class poem of 1899, a fitting farewell to a most enjoyable occasion.

PEN PICTURES OF THE MEETING.

The classes which had reunions were those of '47, '48, '49, '59, '69, '74, '79, '89.

The old classmates who had gathered together in the morning lunched in class groups at the Genesee Hotel.

The most enjoyable features of the day's exercises were the reunion of the old classes, ranging from 1847 to 1889.

After the class reunions the visitors inspected the college buildings and the new departments and the General Hospital.

Two of the curators present were Dr. Thomas D. Strong, '51, of Westfield, and A. G. Ellinwood, '48, of Attica. They are both still young men.

It was hard to say which looked the younger—Dr. Henry Lapp, '65, of Clarence, N. Y., Dr. Theophilus H. Boysen, '74, of Egg Harbor City, N. J., or Dr. H. P. Trull, '68, of Williamsville.

At noon an informal reception to women was held in the college library, followed by a luncheon for the women graduates and the wives and daughters of members of the alumni association.

Dr. W. C. Phelps, the beloved "Uncle Billy" of all students who have attended the university in the last quarter of a century, loomed up during the day and was made much of by his old pupils.

Dr. W. G. Taylor, N. U. '93, did himself proud in the paper he read. He is one of the progressive men of today and reflects credit upon his old alma mater, as well as brings distinction to his new one.

There was mighty little pretense of business at the meetings, the old classmates finding more enjoyment in sitting around and telling stories of events and happenings since leaving college and recalling student days.

William L. Dickinson, of East Saginaw, Michigan, whose diploma will be twenty-one years old next year, was kept busy hunting up classmates of twenty years ago. He found four of 'em and they all got offices.

These gatherings, held in the forenoon, were scattered in every portion of the college building, appropriating recitation and faculty rooms, as well as amphitheaters and library, all of which were placed at their disposal.

Dr. W. H. Bergtold, '87, came all the way from Denver to attend the meeting and read a paper. Dr. Bergtold was formerly city bacteriologist of Buffalo, and, of course, has hosts of friends here, who were mighty glad to see him.

The interest manifested in the reunions of the old classes was general, and a most interesting feature of the day was the fact that nearly every class graduated from the University of Buffalo since it has been in existence was represented.

The earlier classes, those ranging from '47 to '70, were as a matter of course small in number. There were two of the class of '59—Drs. Hamilton R. Smith, of Detroit, and William Warren Potter, of Buffalo. The class of '49 had two members present, that of '79 four.

At the meeting of the '89 class the subject of endowment for laboratory instruction was taken up and before it was dropped $500 had been subscribed. Other classes also considered the subject and the probabilities are that an alumni endowment fund will be the result.

President Lusk, who hails from Warsaw, where there's salt, was a prominent figure at the meeting, which was natural. Dr. Lusk's address was flowery. It could not very well be anything else when one remembers that he spoke from behind bunches of palms and blossoms.

Many of the old students had served time in the government service as surgeons during the misunderstanding of the '60's and they had anecdotes to relate, not having seen each other since the day 'way back when they received that most precious of student possessions—the diploma.

Dr. Walter D. O. K. Strong, of Fishkill-on-the-Hudson, came all the way up from his eastern home to attend the reunion of the class of '49, and then failed to show up at the banquet to respond to his toast. Dr. Strong is one of the liveliest of the young fellows of '49, and when one considers his middle initials one cannot wonder why he went into medicine; he had to, it was foreordained by his parents when they named him.

Dr. Robert P. Bush, '74, of Horseheads, made one of the efforts of his life in his speech at the banquet. Dr. Bush was speaker of the assembly once upon a time and made an admirable record as a statesman. A friend speaking of his personality said: "He's almost as good a statesman as a doctor." Dr. Bush is a serious looking man who combs his hair to the leeward. He has almost as many funny stories at his command as Dr. Fox, of New York. We hope he'll come back again next year.

Dr. George Henry Fox, who came up from New York to show the meeting some landscape effects on syphilis and skin diseases that looked like syphilis, made friends and many of them. Dr. Fox wore a Prince Albert coat, a high hat and told stories like Tom Ochiltree. His story of the Jew who swallowed a $5 gold piece, and who, after being pumped out for an hour or more, could not be induced to give up more than $2.75, had a sort of an old New York Thirteen Club dinner ring. They told the best stories in the world, those old New York Thirteen Club members.

Dr. Wende let Buffalo's death-rate take care of itself for one day and mingled. He was preoccupied until he met Dr. Fox at the depot and had taken him to the college building. They made a fine pair of enjoyable professional men, too. Dr. Wende can tell a story for every one Dr. Fox knows, and Dr. Fox knows more than Chauncey Depew. It isn't hard to imagine that there were not many dull moments when these two were together. Dr. Wende's dinner at the Saturn Club was one of the social features of the day not easily forgotten.

E. N. Brush, of '74, who is at the head of the Moses Sheppard and Enoch Pratt Hospital for the insane, presided as toastmaster at the banquet. He showed how easy it was to be graceful oratorically, and make clean bites of big words. The way he introduced the speakers put them at their ease. That's why the talking was the best part of the banquet. Dr. Brush is a nephew of Mrs. J. F. Miner.

A member of the infant class, anxious for the honor of being an alumnus, and fresh from his books, remarked that "the soup is suffering with fatty degeneration." That shows the evil effects on the mind of plain student fare and pathology. After he's been in practice a few years he will become accustomed to living on the fat of the land.

Hamilton R. Smith, of Detroit, came down from Pingreeville and reunioned with the only other member of the class of '59, who was young enough to get out—Dr. W. W. Potter. Their class reunion was a marvel of parliamentary procedure. There has for a year past been a very well-defined idea that the class of '98 was the wildest bunch of students ever taught to be self-respecting doctors in the University of Buffalo. Dr. Smith told some stories of student days in the '50's, which made a couple of '98 men who had heard them feel that '98 wasn't the only red ink year on the university calendar. Dr. Smith and Dr. Potter, who both served as chief surgeons in the Union army during the '61-5 trouble, entertained a great many friends with stories of military surgery and experience.

Dr. F. F. Hoyer has been practising medicine in Erie County for fifty years, being one of the original '49ers. He is a fund of information. Here is one night's experience in his early days of practice as he told it:

"Well, I remember, in the early '50's, a ride at midnight, with only the stars for a beacon, and a bridle path for my horse. I rode many miles through the woods to my patient and delivered her of fine twins. On my way home I made another call and found seven of the inmates sick with fever. At the next place two of the household had died with cholera, two men were lying on the floor dead drunk, and in another room I found a woman in the last stages of child-birth. In a new country, with many nights and days of hard work like this, a young doctor gets his experience quickly."

---

The most determined and bare-faced effort has recently been made at Albany to inject politics into the educational system of the state by seizing a great many functions of the board of regents. The White bill introduced into the legislature provides for the appointment of a commissioner of education, at $7,000 a year, and he in turn will have the appointment of four deputies, one at $6,000 and three at $5,000 respectively, besides numerous clerks and other department employees. This commissioner is given many of the powers now possessed by the board of regents.

The bill was submitted to the board and the entire body with one exception voted in favor of a resolution condemning the bill as "violent and revolutionary."

The one exception was the present superintendent of education, Charles R. Skinner, who is a regent by virtue of his office. The fact that he cast his vote in favor of the bill would indicate that he had been promised, or at least expected, the position of commissioner.

The sooner the bill is killed, the better. Once let the politicians break into the regents office and they will soon be in control of every department, including that of medicine and then medical education will count for nothing. The man with a "pull" would then be the man who would be certain of a state license to practise medicine.

---

SO FAR the Pan-American directors have not taken up one of the most important things in connection with the welfare of the coming exposition—the sanitary supervision. Nearly everything else has been provided for save this. The designation of a chief sanitary inspector is an absolute necessity and should be taken up at once. Whoever this inspector is, he should be a man of unqualified and unquestioned professional ability.

It may be said that there is plenty of time for this appointment, but to delay naming the man for this very important office would be to do him an injustice whoever he may be. There is a great deal of planning to do. He must of necessity inspect the Pan-American and all the details from the very start; then he will know just what is what and later be in a position to intelligently act.

There will, of course, be plenty of candidates for the place. To avoid friction the JOURNAL would suggest that the Pan-American directors call in Dr. Wende, make him chairman of the health committee and let him suggest the name of the inspector to the executive committee. He is the best situated man in Buffalo to name a thoroughly equipped and proficient physician for the post. And later the inspector should have able assistance. This is a matter of vital importance and should not be deferred until the last moment. It is quite as important a matter as the selection of a site.

---

THE attack on Surgeon-General Sternberg by Dr. Leffingwell has not made any friends for Leffingwell's hobby, but it has strengthened General Sternberg's position.

SURGEON-GENERAL STERNBERG has resolutely fought against political influence in making appointments in his corps. Even the naming of contract surgeons was kept free from such influences and the result has been excellent, honest, scientific work, and Surgeon-General Sternberg's department is the one bureau which is free from taint.

---

THE report of the State Board of Medical Examiners for the examination held January, 1899, exhibits statistics as follows:

| | | |
|---|---|---|
| Total number of candidates | 123 | |
| Successful | 94 | $76\frac{4}{10}$ per cent. |
| Unsuccessful | 29 | $23\frac{6}{10}$ " " |

| | | | |
|---|---|---|---|
| Rejected in anatomy | 10 | Rejected in one topic | 16—16 |
| " " physiology and hygiene, | 2 | " " two topics | 4— 8 |
| " " chemistry | 9 | " " three " | 6—18 |
| " " surgery | 6 | " " four " | 1— 4 |
| " " obstetrics | 5 | " " five " | 1— 5 |
| " " pathology and diagnosis | 19 | " " six " | 1— 6 |
| " " therapeutics, practice and materia medica | 6 | " " seven " | 0— 0 |
| | 57 | | 57 |

| | |
|---|---|
| Passed with honor | 6 |
| Highest general average | $94\frac{9}{10}$ |

The report for the examination held April, 1899, is as follows:

| | | |
|---|---|---|
| Total number of candidates | 68 | |
| Successful | 51 | 75 per cent. |
| Unsuccessful | 17 | 25 " " |

| | | | |
|---|---|---|---|
| Rejected in anatomy | 7 | Rejected in one topic | 8— 8 |
| " " physiology and hygiene, | 3 | " " two topics | 4— 8 |
| " " chemistry | 6 | " " three " | 1— 3 |
| " " surgery | 2 | " " four " | 1— 4 |
| " " obstetrics | 2 | " " five " | 1— 5 |
| " " pathology and diagnosis | 11 | " " six " | 2—12 |
| " " therapeutics, practice and materia medica, | 9 | " " seven " | 0— 0 |
| | 40 | | 40 |

| | |
|---|---|
| Passed with honor | 6 |
| Highest general average | $93\frac{9}{10}$ |

---

UNLESS Buffalo changes in some of its methods of municipal administration it will make a sad showing to the visitors during the exposition year. The streets were never in as bad condition as last winter and the poor department never had greater demands upon it for relief than during the same period. Why the board of public works does not direct the employment of the poor in adequate numbers to keep the streets in good order is beyond the ken of the ordinary observer. Perhaps the ancient mariner at the head of the public works department can tell. He may be a very good administrator of affairs on water, but he sadly fails on land, and singularly enough Buffalo for the most part is built upon land.

Why, too, a suffering public is awakened in the early morning anywhere between 4 and 6 o'clock by the garbage collectors with those rattling trucks is difficult to understand. When the visitors come this will be a fine welcome to offer them! So, again, will the church bell nuisance and the factory whistle pandemonium, the fire tug playing its full share, and contributing its full quota to the latter. These are all relics of a past age and belong to a period of municipal puberty that we hope Buffalo had passed through long ago. They have been complained of time and again, all to no purpose and we fear unless the Pan-American directory takes hold of the matter with insistence that we shall present a sorry showing to our guests. The exposition officers just now exert a great influence and we hope they will begin at the bottom in demanding reforms.

